# Cost consequences analysis of early vocational rehabilitation compared with usual care for stroke survivors

**DOI:** 10.1177/02692155241299372

**Published:** 2024-12-05

**Authors:** Sarah Pyne, Tracey H Sach, Rory Cameron, Helen Risebro, Alexandra Wright-Hughes, Ellen Thompson, Dame Caroline Watkins, Audrey Bowen, Judith Stevens, Amanda J Farrin, Christopher McKevitt, John D Murray, Rory J O’Connor, Julie Phillips, Kate A Radford, on behalf of the RETAKE research group

**Affiliations:** 1 Health Economics Group, 6106 University of East Anglia, Norwich, UK; 2 School of Primary Care, Population Sciences and Medical Education, 7423 University of Southampton, Southampton, UK; 3 National Institute of Health Applied Research Collaboration, East of England, Cambridge, UK; 4 Leeds Institute for Clinical Trials Research, 4468 University of Leeds, Leeds, UK; 5 Applied Health Research Hub and School of Nursing and Midwifery, 6723 University of Central Lancashire, Preston, UK; 6 Manchester Centre for Health Psychology, and Geoffrey Jefferson Brain Research Centre, 5292 University of Manchester, Manchester, UK; 7 PPI, UK; 8 Division of Health and Social Care Research, 4616 King's College London, London, UK; 9 Academic Department of Rehabilitation Medicine, 4468 University of Leeds, Leeds, UK; 10 Centre for Rehabilitation and Ageing Research, School of Medicine, 6123 University of Nottingham, Nottingham, UK

**Keywords:** Stroke, rehabilitation, return to work, cost consequence, economic evaluation

## Abstract

**Objective:**

To compare costs and consequences of Early Stroke Specialist Vocational Rehabilitation (ESSVR) with usual care in working age, stroke survivors over 12 months.

**Design:**

An economic evaluation nested within the pragmatic, multi-centre, randomised, controlled RETurn to work After stroKE (RETAKE) study.

**Setting:**

Twenty-one English and Welsh National Health Service (NHS) hospital-based stroke units. A UK NHS and Personal Social Services perspective was taken in the base-case and a wider perspective (participant, family, employer and other public services) in a secondary analysis.

**Participants:**

A total of 583 stroke survivors age ≥18 years (mean 54.0 years, 69% male).

**Interventions:**

Participants were randomised to ESSVR, an early, individually tailored (in content, dose, intensity and duration) intervention, plus usual care or usual care alone.

**Main measures:**

Disease-specific resource-use data and EQ-5D-5L (health-related quality of life) collected at baseline, 3, 6 and 12 months. Resource-use items were valued using unit costs in UK£ 2021/22. EQ-5D-5L was used to estimate Quality-adjusted life-years (QALYs). If ESSVR was found effective, an incremental cost-utility analysis was planned, otherwise a cost-consequence analysis.

**Results:**

The clinical study found no evidence of a between-group difference in the proportion of participants returning to work at 12 months. This, and the level of missing data, means a cost-consequence analysis is reported. Using imputed data, ESSVR plus usual care is estimated to be more expensive with slightly higher QALYs compared with usual care.

**Conclusions:**

Early Stroke Specialist Vocational Rehabilitation is unlikely to be considered cost-effective over 12 months, which fits with the clinical finding of no between-group difference in return-to-work rates post-stroke.

**Clinical trial registration information:**

The ISRCTN registry: ISRCTN12464275 https://doi.org/10.1186/ISRCTN12464275

## Introduction

Over 100,000 people suffer stroke each year in the UK, and there are 1.3 million stroke survivors. Stroke is most common in people >65 years,^1^ but the incidence amongst those <69 years has increased, accounting for 41% of all strokes.^2^ The economic costs are estimated to be ∼£26 billion per year, of which £8.6 billion (33%) was attributed to UK National Health Service (NHS) and social care costs, £15.8 billion (61%) to unpaid care and £1.6 billion (6%) to lost productivity.^3^ Future incidence, prevalence and costs are anticipated to rise quickly over the next 20 years.^4^

Given the severe consequences of stroke, including physical disabilities, cognitive impairments and emotional changes,^3^ returning to work is challenging. It does, however, help people find purpose and independence and is economically beneficial for society.^5^ Recent Swedish research suggests return-to-work rates amongst those aged 18–58 years improve over several years with 80% back in work at 2 years.^5^

Vocational Rehabilitation helps people find work, prevents job loss and supports career progression despite disability. The need for vocational rehabilitation is recognised in policy and clinical guidelines^6–8^ but is not routinely provided.^9,10^ Only 37% of geographical areas in the UK offered rehabilitation to help stroke survivors return to work.^10^

In a Canadian economic analysis, stroke survivors were less likely to be employed and earned a lower hourly wage than the general population, suggesting that vocational rehabilitation and policies targeting return-to-work could address this.^11^ Whilst there has been growing interest in the economic case for vocational rehabilitation for a number of conditions, and across countries^12–15^ economic evidence relating to vocational rehabilitation post-stroke could not be identified.^16^

The RETurn to work After stroKE (RETAKE) study undertook a within-trial economic evaluation, comparing the costs and quality-adjusted life-years (QALYs) of Early Stroke Specialist Vocational Rehabilitation (ESSVR) plus usual care with usual care alone. Participants were stroke survivors of working age and the perspective was the UK NHS and Personal Social Services in the base-case. A wider perspective (participant, family, employer and other public services, e.g., Department for Work and Pensions) was taken in a secondary analysis.

The objectives were to compare ESSVR with usual care over 12 months by: measuring participant resource-use and estimating costs; estimating participants’ QALYs.

## Methods

The RETAKE study is a pragmatic, multi-centre, randomised controlled trial that compared ESSVR (plus usual care) to usual care alone in adults aged ≥18 years who were in work at stroke onset and admitted to hospital following a new stroke (all severities).^17,18^ Eligible work included ≥2 h of employed, self-employed, paid or voluntary work per week. Registered occupational therapists experienced in delivering specialist stroke rehabilitation in a community setting were preferentially recruited but there were no strict inclusion/exclusion criteria. The RETAKE study aimed to recruit 760 participants from 21 English and Welsh NHS hospital-based stroke units. The nested economic evaluation was performed using individual patient-level data.

Participants were randomised to ESSVR plus usual care or usual care alone sequentially, using a 5:4 allocation ratio to account for the partially nested design.

The intervention, ESSVR, is initiated early, individually tailored (in content, dose, intensity and duration) and seeks to lessen the impact of stroke. It was delivered by specially trained occupational therapists, commenced within 8 weeks of stroke and lasted up to 12 months post-randomisation, with the number of contacts determined by participant need. Meetings also took place with workplace representatives if participants agreed.

Occupational therapists received two days face-to-face training from experienced occupational therapists, and one-day refresher training after six months. Competency was assessed via case vignettes at the end of training and after 12 months of delivering ESSVR through review of case notes. An expert mentor facilitated monthly group mentoring sessions with occupational therapists to provide support, best practice and fidelity. Additional training/mentoring was offered to those that needed it.

Further details are described in the clinical paper.^18^ Patient and public representatives were involved at all stages: questionnaire development, method of data collection, interpretation of results and review of final manuscripts.

### Resource use and costs

Disease-specific resource-use data was collected via a bespoke questionnaire designed for participant self-completion (or with help) at baseline, 3, 6 and 12 months (Supplement 3). The intervention resource use (training, mentoring and delivery) was reported in a questionnaire by the intervention occupational therapists. The number of occupational therapy visits was recorded by occupational therapists, however, to avoid double-counting, we only included participant-reported occupational therapist visits in the economic evaluation. Resource-use information collected included NHS resources (primary care appointments, secondary care in- and out-patient stays, Accident and Emergency attendance and prescriptions), Personal Social Services resources (social workers, health care assistants, stroke-related equipment), Department for Work and Pensions resources (Benefit claims and employment services) and wider resources (participant and carer paid employment [changes to hours worked, time off work], paid home help, other out-of-pocket expenditure). Participants recorded all prescriptions (including type and strength) at baseline, and at subsequent timepoints whether they had stopped/started any medications. The per-participant number of prescriptions could then be calculated (Figure S1). For those that died during the study, data collection points subsequent to date of death were allocated a 0 (not missing).

All costed resource-use items were valued using UK unit costs (in £ Sterling) from 2021/22, with sources and assumptions detailed in Table S1. Time-off work (for participants/carers in paid employment prior to stroke) was costed using the human capital approach and published average wages. Participant and carer out-of-pocket expenses (values) were self-reported by participants in the questionnaire. Prescriptions were valued per time-period using the Prescription Cost Analysis ‘cost per item’ for each month recorded.

Mean (standard deviation [SD]) costs per participant were estimated by study group for each time-period (0–3, 3–6 and 6–12 months) and presented by sector (NHS/Personal Social Services community-based care, NHS secondary care, other Personal Social Services, Department for Work and Pensions and wider costs). A total 12-month NHS and Personal Social Services cost (base-case) was estimated.

### Outcomes

To estimate health-related quality of life, the generic preference-based EQ-5D-5L questionnaire^19^ was combined with mortality data to estimate 12-month QALY score. EQ-5D-5L questionnaire was completed at baseline, 3, 6 and 12 months by the participant (or with help). Responses were converted to utility scores using the mapping model developed by Hernandez-Alava et al. in line with recommendations (model also includes age and sex).^20^ Utility scores of 0 were assigned on date of death; 12-month QALYs were estimated using linear interpolation and area under the curve analysis, adjusting for baseline values.^21^

### Missing data

Missing data is common in randomised controlled trials, particularly when extensive data are collected, which can lead to bias and lack of precision.^22^ As recommended, levels of missing data were reported and the frequency and pattern examined.^22^ Assuming the data is Missing-at-Random, the economic base-case analysis undertook multiple imputation, using chained equations with predictive mean matching to handle the missing cost and outcome data, by treatment group. Variables identified as predictive of missingness (following logistic regression), along with covariates included in the final analysis, were included in the imputation model. Based on up to 75% missing data, there were 75 iterations of imputation^23^ that were pooled using Rubin's rules.^24^

### Economic analyses

This economic evaluation is reported in line with CHEERS guidance.^25^ A Health Economic Analysis Plan was written and followed where possible.^26^ This stated that if clinical benefit was found, the primary analytical approach would be incremental cost-utility analysis: mean (95% confidence interval [CI]) incremental cost and incremental effect (QALY gain) of ESSVR compared with usual care, using seemingly unrelated regression^27^ on multiply imputed data. Regressions would include variables predictive of total costs and outcomes: treatment group, age (years), sex, site and baseline costs/utility score. Results would be presented as Incremental Cost-effectiveness Ratios, with estimates of uncertainty. If no clinical benefit was found or the assumptions of multiple imputation (Missing-at-Random) did not hold, the Health Economic Analysis Plan stated a cost-consequence approach: costs and outcomes presented descriptively.

Two sensitivity analyses and a sub-group analysis were specified in the Health Economic Analysis Plan and were to be undertaken if data allowed. Further details are available online.^26^

Stata MP version 18 was used to conduct analyses. Costs and outcomes were not discounted given the 12-month time frame.

## Results

The randomised controlled trial results, including details of sample size and characteristics, are reported elsewhere.^18^ Of 583 participants (intention-to-treat population) recruited, 324 were randomly assigned to ESSVR and 259 to usual care. Mean age was 54.0 years and similar between groups, but sex differed slightly, with a higher proportion of men in ESSVR compared with usual care (72.8% vs. 64.2%). Trial follow-up completed in June 2023.

### Resource use and costs

Unit costs, together with sources and assumptions are presented in Table S1. Resource-use between groups at baseline was similar (Table S2).

Details of the intervention resource-use per participant are presented in Table S3. The mean cost per participant for training-related intervention was estimated to be £391 (Table 1).

**Table 1.** Mean (SD) and mean (95% CI) difference in resource use/cost (UK£ sterling, 2021/22) over 12-month trial period (participant-reported, available case).

	Resource use			Costs		
Resource item	ESSVR + UC (*n* = 324) Mean ± SD (*n*)	UC (*n* = 259) Mean ± SD (*n*)	Mean difference (95% CI)	ESSVR + UC (*n* = 324) Mean ± SD (*n*)	UC (*n* = 259) Mean ± SD (*n*)	Mean difference (95% CI)
Intervention non-patient contact time (hours) per participant (occupational therapist-reported)^a^						
Trial period (0–12m)	See Table S3 for breakdown of training components.			391 ± 0 (324)	0 ± 0 (259)	-
NHS/PSS resources: Community-based HCP appointments (number of visits/costs)						
0–3m	12.9 ± 21.0 (161)	8.72 ± 14.7 (112)	4.20 (−0.32 to 8.72)	977 ± 1579 (161)	725 ± 1528 (112)	252 (−126 to 629)
3–6m	4.77 ± 7.65 (164)	4.65 ± 9.76 (123)	0.12 (−1.9 to 2.1)	360 ± 630 (164)	284 ± 504 (123)	76 (−60 to 212)
6–12m	3.93 ± 8.70 (148)	2.43 ± 5.13 (104)	1.50 (−0.36 to 3.4)	276 ± 781 (148)	148 ± 400 (104)	128 (−36 to 292)
NHS resources: Secondary care						
Out-patient appointments (number of visits/costs)						
0–3m	4.45 ± 9.78 (174)	2.76 ± 6.28 (123)	1.69 (−0.28 to 3.7)	711 ± 1579 (174)	434 ± 843 (123)	277 (−30 to 584)
3–6m	2.32 ± 4.35 (174)	3.01 ± 10.1 (120)	−0.69 (−2.4 to 1.0)	342 ± 579 (174)	499 ± 1670 (120)	−158 (−429 to 113)
6–12m	1.36 ± 3.17 (155)	1.27 ± 3.15 (101)	0.09 (−0.70 to 0.89)	233 ± 554 (155)	210 ± 458 (101)	23 (−107 to 154)
In-patient stays (number of nights/costs)						
0–3m	2.63 ± 14.9 (181)	5.14 ± 22.0 (131)	−2.51 (−6.6 to 1.6)	1565 ± 8659 (181)	2998 ± 12,312 (131)	−1433 (−3769 to 903)
3–6m	0.38 ± 1.95 (181)	0.33 ± 2.24 (130)	0.04 (−0.43 to 0.51)	301 ± 1508 (181)	333 ± 2310 (130)	−32 (−458 to 395)
6–12m	0.46 ± 3.13 (157)	0.95 ± 5.33 (110)	−0.48 (−1.5 to 0.54)	466 ± 3241 (157)	677 ± 3860 (110)	−212 (−1070 to 647)
A&E attendances (number of visits/costs)						
0–3m	0.22 ± 0.64 (176)	0.13 ± 0.46 (118)	0.09 (−0.04 to 0.23)	54 ± 156 (176)	31 ± 112 (118)	23 (−9.9 to 56)
3–6m	0.13 ± 0.47 (168)	0.16 ± 0.52 (117)	−0.04 (−0.16 to 0.08)	30 ± 113 (168)	39 ± 127 (117)	−9 (−37 to 19)
6–12m	0.23 ± 0.79 (137)	0.22 ± 0.61 (101)	0.008 (−0.18 to 0.19)	55 ± 190 (137)	53 ± 148 (101)	2 (−43 to 47)
NHS resources: Prescription medications per participant (number of medications prescribed/costs)						
0–3m	2.90 ± 2.83 (176)	2.96 ± 3.51 (132)	−0.06 (−0.77 to 0.65)	60 ± 183 (176)	80 ± 395 (132)	−20 (−86 to 46)
3–6m	3.26 ± 3.12 (136)	3.17 ± 3.79 (89)	0.10 (−0.82 to 1.01)	69 ± 206 (136)	99 ± 477 (89)	−30 (−121 to 61)
6–12m	3.42 ± 3.13 (99)	3.25 ± 3.79 (63)	0.17 (−0.91 to 1.25)	124 ± 302 (99)	242 ± 1119 (63)	−118 (−352 to 116)
PSS resources: Other^b^						
Stroke-related equipment (number of items/costs)						
	No./*n* (%)	No./*n* (%)				
0–3m	51/183 (28%)	30/133 (23%)	-	-	-	-
3–6m	22/181 (12%)	10/127 (7.8%)	-	-	-	-
6–12m	15/154 (9.7%)	8/108 (7.4%)	-	-	-	-
Department for Work and Pensions resources						
Benefits (number claiming a state benefit/costs)						
0–3m	51/173 (29%)	30/122 (25%)	-	322 ± 582 (170)	274 ± 569 (117)	48 (−89 to 184)
3–6m	57/177 (32%)	33/125 (26%)	-	373 ± 626 (172)	320 ± 589 (124)	53 (−88 to 200)
6–12m	57/160 (36%)	27/110 (25%)	-	890 ± 1380 (157)	549 ± 1130 (110)	340 (27 to 650)
Employment services (contacts with employment services/costs)						
	Mean ± SD (*n*)	Mean ± SD (*n*)				
0–3m	0.27 ± 0.85 (175)	11.9 ± 44.3 (123)	1.43 (−8.5 to 11.4)	13 ± 42 (172)	12 ± 44 (123)	1 (−9 to 11)
3–6m	0.22 ± 0.97 (174)	0.14 ± 0.58 (122)	0.08 (−0.11 to 0.28)	11 ± 47 (174)	9 ± 35 (122)	2 (−8 to 12)
6–12m	0.38 ± 1.78 (153)	0.08 ± 0.61 (109)	0.30 (−0.05 to 0.65)	19 ± 87 (153)	4 ± 40 (108)	14 (−3 to 32)
Wider resources: out-of-pocket^c^						
Time off work (net change in hours/costs)^d^						
0–3m	290 ± 272 (150)	213 ± 259 (105)	76.2 (9.4 to 143)	5220 ± 4900 (150)	3840 ± 4660 (105)	1370 (170 to 2600)
3–6m	119 ± 230 (151)	87.6 ± 194 (106)	31.1 (−23 to 85)	2140 ± 4130 (151)	1580 ± 3490 (106)	560 (−410 to 1500)
6–12m	185 ± 389 (137)	107 ± 298 (101)	78.1 (−13 to 170)	3340 ± 7010 (137)	1930 ± 5370 (101)	1410 (−240 to 3100)
Unpaid carer time off work (net change in hours/costs)						
0–3m	−6.83 ± 29.0 (71)	−9.64 ± 9.64 (50)	2.8 (−26.7 to 32.3)	123 ± 553 (71)	174 ± 218 (59)	−51 (−580 to 480)
3–6m	−6.08 ± 71.4 (92)	−1.92 ± 27.4 (65)	−4.15 (−23 to 14)	109 ± 1290 (92)	34.6 ± 495 (65)	75 (−260 to 410)
6–12m	3.11 ± 36.5 (115)	−12.7 ± 62.97 (80)	15.8 (1.77 to 29.9)	−56 ± 658 (115)	229 ± 1130 (80)	−285 (−538 to −32)
Paid home help (number receiving visits)^e^						
0–3m	11/181 (6.1%)	12/132 (9.1%)	-	133 ± 877 (176)	95.3 ± 584 (129)	37.7 (−140 to 210)
3–6m	12/181 (6.6%)	13/127 (10%)	-	117 ± 785 (178)	249 ± 1400 (125)	−133 (−380 to 120)
6–12m	3/159 (1.9%)	5/111 (4.5%)	-	7 ± 66 (158)	204 ± 1860 (110)	−197 (−490 to 95)
Out-of-pocket expenditure items (number of items)^e^						
0–3m	79/180 (44%)	56/132 (42%)	-	118 ± 587 (175)	38 ± 298 (131)	79 (−31 to 190)
3–6m	59/177 (33%)	40/125 (32%)	-	67 ± 365 (174)	35 ± 102 (118)	32 (−36 to 100)
6–12m	43/155 (28%)	28/112 (25%)	-	189 ± 947 (152)	76 ± 288 (110)	113 (−71 to 300)
Non-NHS/PSS support services (number received at least one service)^e^						
0–3m	32/181 (18%)	19/130 (15%)	-	0.34 ± 2.50 (181)	0.88 ± 5.29 (130)	−0.55 (−1.44 to 0.34)
3–6m	27/181 (15%)	19/127 (15%)	-	0.09 ± 1.05 (181)	1.96 ± 21.38 (126)	−1.87 (−5.00 to 1.26)
6–12m	26/155 (17%)	13/110 (12%)	-	19.79 ± 241.75 (154)	6.18 ± 49.68 (110)	13.61 (−32.48 to 59.70)

CI: confidence interval; NHS: UK National Health Services; PSS: Personal Social Services; SD: standard deviation; UC: usual care.

a Details informed by Radford et al. 2024.^28^

b Stroke-related equipment not costed due to small numbers and lack of adequate detail.

c Not included in the base-case analysis.

d Includes changes to hours worked/time off work for those in paid employment prior to stroke.

e Items reported as total out-of-pocket expenditure for participants/family and friends.

To reduce loss of information due to missing data, mean resource-use and associated costs are presented by trial period (0–3, 3–6 and 6–12 months) and treatment group (Table 1). Looking at NHS/Personal Social Services resource use and related costs, there was no significant difference between treatment groups in any trial period for any resource item. There were a greater number of community-based HCP visits (not significant) for ESSVR compared with usual care, resulting in slightly higher costs. This may reflect the intervention-occupational therapist appointments. A breakdown of community-based HCP visits is in Table S4, where the difference in occupational therapist appointments can be seen (6.28 [SD 7.76, *n* = 93] ESSVR vs. 4.75 [SD 9.42, *n* = 63] usual care). The only significant difference in community-based HCP visits was for physiotherapist (4.98 [SD 13.81, *n* = 97] ESSVR vs. 1.05 [SD 2.37, *n* = 66] usual care). A breakdown of secondary care contacts is in Table S5. Out-patient occupational therapist appointments were very similar between treatment groups (0.92 [SD 2.87, *n* = 101] ESSVR vs. 1.1 [SD 3.58, *n* = 60] usual care). It was not possible to cost data collected on stroke-related equipment due to small numbers and inadequate reporting.

Total NHS and Personal Social Services costs are presented in Table 2. In the complete case analysis, only 86/583 (15%) of participants remain. The mean difference in costs were less for ESSVR compared with usual care (£5145 vs. £7832).

**Table 2.** Participant mean (SD) and mean (95% CI) difference in costs (£, NHS/PSS) and outcomes over 12-month trial period (available case, unless stated otherwise).

	Mean ± SD (*n*)	Mean ± SD (*n*)	Mean difference (95% CI)
	ESSVR + UC (*n* = 324)	UC (*n* = 259)	
Costs (unadjusted)^b^			
Total at baseline	481 ± 3596 (216)	703 ± 3385 (185)	−222 (−912 to 467)
Total at 3m	2568 ± 6281 (135)	4468 ± 14,057 (92)	−1899 (−4608 to 810)
Total at 6m	1150 ± 2246 (111)	1602 ± 4033 (69)	−452 (−1376 to 472)
Total at 12m	1065 ± 2694 (82)	1213 ± 4586 (50)	−148 (−1401 to 1104)
Total trial period	4754 ± 6733 (57)	7832 ± 7961 (29)	−3077 (−7818 to 1663)
Total trial period inc. intervention	5145 ± 6733 (57)	7832 ± 7961 (29)	−2686 (−7426 to 2055)
Costs (adjusted)^a^			
Total trial period inc. intervention (MI)	(324)	(259)	1282 (−933 to 3497)
Utility score/QALYs (unadjusted)			
Utility score (EQ-5D-5L) at baseline	0.589 ± 0.285 (315)	0.590 ± 0.308 (245)	−0.001 (−0.050 to 0.049)
Utility score (EQ-5D-5L) at 3m	0.632 ± 0.261 (191)	0.669 ± 0.248 (143)	−0.037 (−0.092 to 0.019)
Utility score (EQ-5D-5L) at 6m	0.679 ± 0.238 (189)	0.677 ± 0.256 (133)	0.002 (−0.052 to 0.057)
Utility score (EQ-5D-5L) at 12m	0.674 ± 0.267 (167)	0.682 ± 0.264 (122)	−0.007 (−0.070 to 0.055)
12-month QALY score	0.699 ± 0.189 (103)	0.685 ± 0.227 (64)	0.014 (−0.050 to 0.078)
QALYs (adjusted)^a^			
12-month QALY score (MI)	(324)	(259)	0.021 (−0.011 to 0.052)

CI: confidence interval; MI: multiply imputed; NHS: UK National Health Services; QALY: quality-adjusted life-years; SD: standard deviation; UC: usual care.

a Seemingly unrelated regression analysis, adjusted for age, sex, utility/costs at baseline and site.

b Includes NHS/PSS resource use: community-based HCP appointments, out-patient appointments, in-patient stays, A&E attendance and prescription medication use.

With respect to wider, out-of-pocket, resource use (outside NHS/ Personal Social Services or Department for Work and Pensions), there were some differences (Table 1), but small numbers reporting. Time taken off work by participants post-stroke was greater for ESSVR than for usual care in all time periods and significantly so in the 0–3 month period (290 vs. 213 h, respectively), translating into greater costs for ESSVR. A significant difference in unpaid carer time off work was estimated for the 6–12-month period (3.11 h ESSVR vs. −12.7 h usual care), but only 8/195 complete responses given, reported a change in carer work hours. Negative hours, reported for usual care, indicate that at least one carer increased their hours. There was little between group difference for other wider (outside NHS/Personal Social Services) resource-use items.

Although state benefits represent a transfer payment, we recorded information on these to measure any impact. Nominally higher levels of state benefit claims were made in ESSVR compared with usual care (Table 1), which translated into significantly higher costs (£890 vs. £549, respectively) in the 6–12-month period. Contact with employment services did not differ between groups at any timepoint.

### Outcomes

Table 2 presents utility scores at all time-points and 12-month QALY scores (adjusted and unadjusted). Utility scores were similar between groups at all time-points, including baseline, resulting in a similar 12-month QALY score (0.699 ESSVR vs. 0.685 usual care). However, there was an increasing proportion of missing data so this is based on less than 1/3 of patients randomised (32% ESSVR, 25% usual care).

### Missing data

The proportion of missing data is reported in Table 3. At all time-points levels were high for both resource use and measures of health-related quality of life – 85% missing for total costs and 71% missing for total QALYs (complete case analysis) – with implications for the results of imputation. Exploration revealed no clear evidence of participants progressively failing to complete questionnaires. Logistic regression of missingness (costs or QALYs) on randomisation group, stratification variables and other baseline variables (see Table S6) demonstrated missing cost data was associated only with treatment group (more missing for usual care than ESSVR) whilst missing QALY data was associated with education level (more missing with lower education levels) and a single study site had significantly less QALY data. While not conclusive, Missing-at-Random is the most likely mechanism of missingness and multiple imputation using the methods described are appropriate. The following variables were included in imputation: treatment group, age, sex, site, education level, utility and costs at baseline, 3, 6 and 12 months.

**Table 3.** Proportion of missing values (survivor data).

Variable	ESSVR + UC (*n* = 324)	UC (*n* = 259)	Total (*n* = 583)
Baseline variables			
Treatment allocation	0	0	0
Age	0	0	0
Sex	1/324 (0.3%)	2/259 (0.8%)	3/583 (0.5%)
Utility score at baseline	9/324 (2.8%)	14/324 (4.3%)	23/583 (3.9%)
Resource use at baseline^a^	108/324 (33%)	74/259 (29%)	182/583 (31%)
Cost variables			
Resource use at 3 months^a^	189/324 (58%)	167/259 (64%)	356/538 (61%)
Resource use at 6 months^a^	213/324 (66%)	190/259 (73%)	403/583 (69%)
Resource use at 12 months^a^	242/324 (75%)	209/259 (81%)	451/583 (77%)
Outcome variables for health-related quality of life			
Utility score at 3 months	133/324 (41%)	116/259 (45%)	249/583 (43%)
Utility score at 6 months	135/324 (42%)	126/259 (49%)	261/583 (45%)
Utility score at 12 months	157/324 (49%)	137/259 (53%)	294/583 (50%)
Outcomes for cost-utility analysis			
Total costs	267/324 (82%)	231/259 (89%)	498/583 (85%)
Total QALYs	221/324 (68%)	195/259 (75%)	416/583 (71%)

QALY: quality-adjusted life-years; UC: usual care.

a Includes NHS/PSS resource use: community-based HCP appointments, out-patient appointments, in-patient stays, A&E attendance and prescription medication use.

### Economic analyses

Also presented in Table 2 is the adjusted (for age, sex, baseline utility/costs and site) total costs and 12-month QALY score, using imputed data. The mean difference (95% CI) in total costs swapped over in favour of usual care to £1282 (−£933 to £3497) ESSVR vs. usual care, likely due to the significant association between treatment group and missing cost data. The mean difference (95% CI) in 12-month QALY score was 0.021 (−0.011 to 0.052) ESSVR vs. usual care.

## Discussion

The multiply imputed data suggest ESSVR is more expensive per participant than usual care but also slightly more effective in terms of QALYs (health-related quality and quantity of life). We did not undertake an incremental cost-utility analysis for reasons detailed in the Health Economic Analysis Plan but had an Incremental Cost-effectiveness Ratio been estimated it would be considerably above the willingness-to-pay thresholds typically used by NICE.^29^ These results fit with the clinical findings and are indicative of recruited participants having less severe strokes and the return-to-work rate in the usual care group being more than double that observed in the feasibility work that informed the sample size estimate for the full study. COVID also changed work norms in ways that possibly helped return-to-work. Nevertheless, this study adds useful evidence for a topic that has not received research attention before. It indicates the scale of resources used in the aftermath of a stroke from different perspectives (albeit impacted by COVID) and the impact on health-related quality of life. The levels of community-based and secondary care visits reported for usual care were surprising because they were similar to ESSVR and because the levels were greater than observed in the process evaluation embedded case studies.^30^ Occupational therapy visits for usual care may have been misreported if participants were uncertain about which type of professional they saw.

A strength of this study is that it uses data gathered alongside a large randomised controlled trial seeking to address an important question about the effectiveness and cost-effectiveness of vocational rehabilitation for individuals who have recently experienced stroke. This has generated new insight into the health services used and impact on health-related quality of life for people post-stroke.

A limitation of the study is that questionnaire completion rates were low and declined over time, resulting in significant amounts of missing data. This suggests questionnaires were too burdensome for these patients (evidence of an association between stroke severity and missingness was found in the clinical results^18^) and needed to be reduced in length by focusing more carefully on the likely cost drivers or other data collection methods used. The pilot work for RETAKE did explore resource-use data collection methods, including notes review, but it was clear that no single source of data would have captured the full range of relevant resources.

A further limitation is the restricted time horizon of the analysis. Had vocational rehabilitation been found effective it is likely the impact of helping people return to work would have significant impacts beyond 12 months. Indeed, there is evidence that suggests return-to-work can take years.^5^ A decision was taken at the outset not to develop a longer-term economic model but to focus on within-trial cost-effectiveness and given the findings, it is unlikely extending the time frame in an economic model would change the results.^5^

While some interesting observations were made within the data, for example, higher physiotherapist appointments for ESSVR vs. usual care, it is important to consider the multiple comparison problem,^31^ which states that the greater the number of inferences made, the greater the chance of a false positive. Occupational therapists delivering ESSVR, likely facilitated referrals as part of the ESSVR case-coordinated approach, corroborated by the process evaluation,^30^ or participants mistook occupational therapy appointments for other HCPs, but it is hard to draw conclusions.

COVID occurred during this trial, which not only changed work norms, for example, facilitating home-based and flexible working but also affected the use of health and other services due to differing priorities and restrictions. Consequently, the findings in this paper reflect this context and may not generalise to a post-COVID context. In a deviation from the Health Economic Analysis Plan, we did not undertake planned sub-group analyses by COVID infection, furlough time-period or by ‘good intervention compliers’ due to the levels of missing data. Likewise, we were only able to undertake partial (wider perspective, carer perspective, complete case), or in some cases no (threshold analysis or analysis excluding outliers), planned sensitivity analyses.

The clinical results suggest there may be benefit from ESSVR for older and more severely affected people post-stroke. Further research exploring the potential value-for-money of ESSVR in these sub-groups may, therefore, be worthwhile.^18^ The economic value of a more light-touch approach, for example, a self-guided online toolkit, to aid return-to-work for those experiencing mild-to-moderate strokes, would also be worthwhile, as the UK is currently experiencing rising numbers of economically inactive individuals aged >50 years due to long-term illness.^32^

Further research to explore how to maximise resource-use data capture in this patient population across relevant sectors would be useful for informing the design of future trials.

In conclusion, the slight improvement in QALYs is unlikely to justify the higher costs of ESSVR (as delivered in this trial for this population) compared with usual care over 12 months. However, there is much uncertainty around this conclusion, given the levels of missing data and lack of longer-term evidence.

**Clinical messages**

- Early Stroke Specialist Vocational Rehabilitation (ESSVR) delivered small improvements in health-related quality of life that did not justify the increased costs over 12 months.
- Value-for-money could be explored in older and more severely affected stroke survivors and over a longer time frame.
- Transferability of these results to clinical practice should consider COVID, which impacted return-to-work.

## Supplemental Material

sj-pdf-1-cre-10.1177_02692155241299372 - Supplemental material for Cost consequences analysis of early vocational rehabilitation compared with usual care for stroke survivors Supplemental material, sj-pdf-1-cre-10.1177_02692155241299372 for Cost consequences analysis of early vocational rehabilitation compared with usual care for stroke survivors by Sarah Pyne, Tracey H Sach, Rory Cameron, Helen Risebro, Alexandra Wright-Hughes, Ellen Thompson, Dame Caroline Watkins, Audrey Bowen, Judith Stevens, Amanda J Farrin, Christopher McKevitt, John D Murray and Rory J O’Connor, Julie Phillips, Kate A Radford, in Clinical Rehabilitation

sj-pdf-2-cre-10.1177_02692155241299372 - Supplemental material for Cost consequences analysis of early vocational rehabilitation compared with usual care for stroke survivors Supplemental material, sj-pdf-2-cre-10.1177_02692155241299372 for Cost consequences analysis of early vocational rehabilitation compared with usual care for stroke survivors by Sarah Pyne, Tracey H Sach, Rory Cameron, Helen Risebro, Alexandra Wright-Hughes, Ellen Thompson, Dame Caroline Watkins, Audrey Bowen, Judith Stevens, Amanda J Farrin, Christopher McKevitt, John D Murray and Rory J O’Connor, Julie Phillips, Kate A Radford, in Clinical Rehabilitation

sj-pdf-3-cre-10.1177_02692155241299372 - Supplemental material for Cost consequences analysis of early vocational rehabilitation compared with usual care for stroke survivors Supplemental material, sj-pdf-3-cre-10.1177_02692155241299372 for Cost consequences analysis of early vocational rehabilitation compared with usual care for stroke survivors by Sarah Pyne, Tracey H Sach, Rory Cameron, Helen Risebro, Alexandra Wright-Hughes, Ellen Thompson, Dame Caroline Watkins, Audrey Bowen, Judith Stevens, Amanda J Farrin, Christopher McKevitt, John D Murray and Rory J O’Connor, Julie Phillips, Kate A Radford, in Clinical Rehabilitation

sj-pdf-4-cre-10.1177_02692155241299372 - Supplemental material for Cost consequences analysis of early vocational rehabilitation compared with usual care for stroke survivors Supplemental material, sj-pdf-4-cre-10.1177_02692155241299372 for Cost consequences analysis of early vocational rehabilitation compared with usual care for stroke survivors by Sarah Pyne, Tracey H Sach, Rory Cameron, Helen Risebro, Alexandra Wright-Hughes, Ellen Thompson, Dame Caroline Watkins, Audrey Bowen, Judith Stevens, Amanda J Farrin, Christopher McKevitt, John D Murray and Rory J O’Connor, Julie Phillips, Kate A Radford, in Clinical Rehabilitation

sj-pdf-5-cre-10.1177_02692155241299372 - Supplemental material for Cost consequences analysis of early vocational rehabilitation compared with usual care for stroke survivors Supplemental material, sj-pdf-5-cre-10.1177_02692155241299372 for Cost consequences analysis of early vocational rehabilitation compared with usual care for stroke survivors by Sarah Pyne, Tracey H Sach, Rory Cameron, Helen Risebro, Alexandra Wright-Hughes, Ellen Thompson, Dame Caroline Watkins, Audrey Bowen, Judith Stevens, Amanda J Farrin, Christopher McKevitt, John D Murray and Rory J O’Connor, Julie Phillips, Kate A Radford, in Clinical Rehabilitation

sj-docx-6-cre-10.1177_02692155241299372 - Supplemental material for Cost consequences analysis of early vocational rehabilitation compared with usual care for stroke survivors Supplemental material, sj-docx-6-cre-10.1177_02692155241299372 for Cost consequences analysis of early vocational rehabilitation compared with usual care for stroke survivors by Sarah Pyne, Tracey H Sach, Rory Cameron, Helen Risebro, Alexandra Wright-Hughes, Ellen Thompson, Dame Caroline Watkins, Audrey Bowen, Judith Stevens, Amanda J Farrin, Christopher McKevitt, John D Murray and Rory J O’Connor, Julie Phillips, Kate A Radford, in Clinical Rehabilitation

## Data Availability

Data supporting this work are available on reasonable request. All requests will be reviewed by relevant stakeholders, based on the principles of a controlled access approach. Requests to access data should be made to CTRU-DataAccess@leeds.ac.uk in the first instance.
