# Supplementary material for: Cost consequences analysis of early vocational rehabilitation compared with usual care for stroke survivors
Source: Clin Rehabil. 2024 Dec 5;39(2):161–73. doi: 10.1177/02692155241299372 (PMC11846267; doi:10.1177/02692155241299372)
Supplement: sj-pdf-1-cre-10.1177_02692155241299372 - Supplemental material for Cost consequences analysis of early vocational rehabilitation compared with usual care for stroke survivors [file sj-pdf-1-cre-10.1177_02692155241299372.pdf]

Supplementary material 1: Additional tables and figures

Table S1: Unit costs used to value resource use (UK£ sterling, 2021/22)

Cost Item	Unit Cost (£)	Assumptions
Intervention		
ESSVR training: workshop facilitator (per hour)	75[1]	Principal occupational therapist (more experienced)
ESSVR training: workshop attendance (per hour)	47[1]	Community occupational therapist (local authority)
ESSVR training: competency assessment (per hour)	47[1]	Community occupational therapist (local authority)
ESSVR mentoring: facilitator (per hour)	75[1]	Principal occupational therapist (more experienced)
ESSVR mentoring: attendance (per hour)	47[1]	Community occupational therapist (local authority)
ESSVR face-to-face contact (per hour)	47[1]	Community occupational therapist (local authority)
Primary care appointments (per visit)		
Occupational therapist	99[2]	Adult one-to-one session
General practitioner (GP)	35[1]	Surgery consultation lasting 9.22 min. Inc direct staff costs. Exc qualifications
Practice Nurse	15[1,3]	Surgery consultation lasting 15.5 min. Inc direct staff costs. Exc qualifications
District nurse	54[2]	Adult, face-to-face session
Case manager	77[2]	Other therapist, adult, one-to-one
Counsellor	77[2]	Other therapist, adult, one-to-one
Speech and language therapist (SLT)	128[2]	Adult, one-to-one
Physiotherapist	73[2]	Adult, one-to-one
Social worker	112[1,4]	40 min appointment. Inc direct staff costs. Exc qualifications
Rehabilitation assistant	131[2]	Stroke community rehabilitation team
Health care assistant	14[1,5,6]	Face-to-face day-time week price. 30 min visit based on majority
Walk-in centre	82[2]	NHS walk-in centres: weighted average of all entries.
Other	35[1]	Based on a GP (common) visit (as above)
Secondary care: out-patient appointments (per visit)		
Consultant	185[2]	Average of all consultant-led
Psychiatrist	231[2]	Liaison psychiatry service
Physiotherapist	100[2]	Physiotherapy service
Speech and language therapist (SLT)	188[2]	Speech and language therapy service
Nurse	76[2]	Other specialist nursing, adult, face-to-face

Stroke team	302[2]	Stroke medicine service
Occupational therapist	99[2]	Occupational therapist, adult, one-to-one
Radiologist	117[2]	
Ophthalmologist	142[2]	
Cardiac & pulmonary tests	41[2]	
Blood tests	7[2]	
Other	165[2]	
Secondary care: in-patient stays		
Neurology ward	533[2]	
Other ward	1038[2]	
Accident and Emergency	242[2]	
Prescription medications		
Various (average cost)	4[7]	Assume one prescription per month, applied 'cost per item'. If ambiguous, used most prescribed closest match.
Social services resources		
Stroke- related equipment	n/a	
Wider resources: out-of-pocket		
Employment services	49[8]	Assume costs are same as disability employment advisor[8]
Time off work (hourly)	18[9]	
Unpaid carer (hourly)	18[9]	
Wider resources: out-of-pocket		
Employment and Support Allowance (ESA)	91[10]	
Personal Independence Payment (PIP)	73[10]	
Universal Credit	393[10]	
Statutory Sick Pay (SSP)	117[10]	
Other	73[10]	

Table S2: Mean (sd) resource use and mean (95% CI) difference in resource use at baseline (over 6 weeks prior to stroke) (Participant reported, available case)

Resource item	Resource item statistic	ESSVR + usual care (n=324)	Usual care (n=259)	Mean difference (95% CI)
NHS/PSS resources: Community-based HCP appointments (number of visits)				
Occupational therapist	Mean ± sd (n)	0.01 ± 0.14 (234)	0.01 ± 0.07 (201)	0.01 (-0.01 to 0.03)
General practitioner	Mean ± sd (n)	0.53 ± 0.99 (240)	0.42 ± 0.83 (200)	0.11 (-0.06 to 0.29)
Practice Nurse	Mean ± sd (n)	0.12 ± 0.40 (242)	0.27 ± 0.83 (201)	-0.14 (-0.26 to -0.03)
District nurse	Mean ± sd (n)	0 ± 0 (243)	0.07 ± 0.86 (202)	-0.07 (-0.18 to 0.03)
Case manager	Mean ± sd (n)	0 ± 0 (243)	0 ± 0 (202)	0 (-)
Counsellor	Mean ± sd (n)	0.06 ± 0.46 (242)	0.01 ± 0.07 (202)	0.06 (-0.01 to 0.12)
SLT	Mean ± sd (n)	0 ± 0 (243)	0 ± 0 (202)	0 (-)
Physiotherapist	Mean ± sd (n)	0.02 ± 0.17 (243)	0.02 ± 0.23 (202)	-0.00 (-0.04 to 0.03)
Social worker	Mean ± sd (n)	0 ± 0 (243)	0 ± 0 (202)	0 (-)
Rehabilitation assistant	Mean ± sd (n)	0.02 ± 0.38 (243)	0 ± 0 (202)	0.02 (-0.03 to 0.08)
Health care assistant	Mean ± sd (n)	0 ± 0 (243)	0 ± 0 (200)	0 (-)
Walk-in centre	Mean ± sd (n)	0.05 ± 0.42 (243)	0.03 ± 0.20 (202)	0.02 (-0.04 to 0.09)
Other	Mean ± sd (n)	0.06 ± 0.32 (244)	0.05 ± 0.26 (202)	0.02 (-0.04 to 0.08)
Total	Mean ± sd (n)	0.89 ± 1.56 (239)	0.87 ± 1.65 (198)	0.02 (-0.28 to 0.32)
NHS resources: out-patient appointments (number of visits)				
Consultant	Mean ± sd (n)	0.14 ± 0.51 (311)	0.13 ± 0.47 (246)	0.00 (-0.08 to 0.08)
Occupational therapist	Mean ± sd (n)	0.00 ± 0.06 (316)	0 ± 0 (0)	0.00 (-0.00 to 0.01)
Psychologist	Mean ± sd (n)	0 ± 0 (316)	0 ± 0 (248)	0 (-)
Physiotherapist	Mean ± sd (n)	0.01 ± 0.18 (316)	0.02 ± 0.16 (248)	-0.00 (-0.03 to 0.02)
SLT	Mean ± sd (n)	0 ± 0 (316)	0 ± 0 (248)	0 (-)
Nurse	Mean ± sd (n)	0.03 ± 0.41 (316)	0.01 ± 0.09 (248)	0.02 (-0.00 to 0.08)
Stroke team	Mean ± sd (n)	0.00 ± 0.06 (316)	0.00 ± 0.06 (248)	-0.00 (-0.00 to 0.01)
Radiology	Mean ± sd (n)	0.02 ± 0.16 (315)	0.02 ± 0.21 (248)	-0.00 (-0.03 to 0.03)
Ophthalmology	Mean ± sd (n)	0.02 ± 0.15 (315)	0.04 ± 0.25 (248)	-0.02 (-0.05 to 0.01)
Cardiac/pulmonary tests	Mean ± sd (n)	0 ± 0 (316)	0.00 ± 0.06 (248)	-0.00 (-0.01 to 0.002)
Blood tests	Mean ± sd (n)	0.01 ± 0.08 (316)	0.01 ± 0.13 (248)	-0.00 (-0.02 to 0.02)
Other	Mean ± sd (n)	0.06 ± 0.46 (315)	0.02 ± 0.16 (248)	0.04 (-0.02 to 0.10)
Total	Mean ± sd (n)	0.30 ± 1.00 (309)	0.25 ± 0.75 (246)	0.05 (-0.10 to 0.20)
NHS resources: in-patient stays (number of nights)				
Stroke ward	Mean ± sd (n)	0.16 ± 2.81 (317)	0.04 ± 0.45 (253)	0.11 (-0.24 to 0.47)
Neuro/stroke rehab unit	Mean ± sd (n)	0 ± 0 (317)	0 ± 0 (253)	0 (-)
ICU/HDU	Mean ± sd (n)	0.07 ± 1.29 (317)	0 ± 0 (253)	0.07 (-0.09 to 0.23)
Other ward	Mean ± sd (n)	0.05 ± 0.35 (316)	0.45 ± 2.80 (253)	-0.40 (-0.72 to -0.09)
Total	Mean ± sd (n)	0.28 ± 3.11 (316)	0.50 ± 2.98 (253)	-0.22 (-0.72 to 0.29)
NHS resources: Other				

A&E attendances (visits)	Mean ± sd (n)	0.09 ± 0.33 (310)	0.07 ± 0.31 (245)	0.02 (-0.03 to 0.07)
Prescription medications (number prescribed)	Mean ± sd (n)	1.99 ± 2.43 (313)	2.33 ± 3.01 (252)	-0.34 (-0.79 to 0.11)
PSS resources: other				
Stroke-related equipment (no. of items)	No./n (%)	1/310 (0.3%)	3/248 (1.2%)	-0.01 (-0.02 to 0.01)
Department for Work and Pensions resources				
State benefits (no. claiming)	No./n (%)	30/303 (9.9%)	22/241 (9.1%)	-
Employment services (no. of contacts)	Mean ± sd (n)	0.02 ± 0.25 (313)	0.01 ± 0.13 (247)	0.01 (-0.02 to 0.05)
Wider resources: out-of-pocket[†]				
Time off work (hrs)	Mean ± sd (n)	18.2 ± 72.0 (294)	18.9 ± 61.5 (226)	-0.71 (-12 to 11)
Unpaid carer time off work (hrs)	Mean ± sd (n)	0.96 ± 15.8 (316)	0.13 ± 2.01 (253)	0.84 (-1.13 to 2.80)
Paid home help (no. of visits)	No./n (%)	10/316 (3.1%)	7/250 (2.8%)	-
Out-of-pocket expenditure (no. items)[§]	No./n (%)	25/312 (8.0%)	17/249 (6.8%)	-
Non-NHS/PSS support services (no. received)[§]	No./n (%)	1/312 (0.3%)	5/248 (2.0%)	-

CI, confidence interval; HDU, high dependency unit; ICU, intensive care unit; SLT, Speech and language therapist; PSS, personal social services; sd, standard deviation.

[‡] Includes both time off work, and reduced hours for those in paid employment prior to stroke (since number of working hours at baseline required to calculate change in workload)

[§] These items were reported as total out-of-pocket expenditure for participants

Table S3: Intervention non-patient contact time (hours) per participant (OT-reported): treatment period (0–12m)*

Resource item	Statistics	ESSVR + usual care (n=324)	Usual care (n=259)	Mean difference (95% CI)
ESSVR initial training session – OT time	Total attendees<br>Mean (sd)	49<br>2.27 ± 0 (324)	0<br>0 ± 0 (259)	-
ESSVR initial training session – mentor time	Total sessions<br>Mean (sd)	17<br>0.79 ± 0 (324)	0<br>0 ± 0 (259)	-
ESSVR initial training session – assessment marking (mentor time)	Total assessed<br>Mean (sd)	49<br>0.11 ± 0 (324)	0<br>0 ± 0 (259)	-
ESSVR refresher training session – OT time	Total attendees<br>Mean (sd)	49<br>1.13 ± 0 (324)	0<br>0 ± 0 (259)	-
ESSVR refresher training session – mentor time	Total sessions<br>Mean (sd)	7<br>0.16 ± 0 (324)	0<br>0 ± 0 (259)	-
ESSVR telephone/online mentoring – OT time	Total calls<br>Mean (sd)	728<br>1.80 ± 0 (324)	0<br>0 ± 0 (259)	-
ESSVR telephone/online mentoring – mentor time	Total calls<br>Mean (sd)	239<br>0.74 ± 0 (324)	0<br>0 ± 0 (259)	-
ESSVR employer contact – OT time	Total contacts<br>Mean (sd)	109<br>0.08 ± 0 (324)	0<br>0 ± 0 (259)	-
ESSVR employer visit – OT time	Total visits<br>Mean (sd)	239<br>0.17 ± 0 (324)	0<br>0 ± 0 (259)	-

CI, confidence interval; OT, occupational therapist; sd, standard deviation.

*OT-recorded face-to-face contact not included, to prevent double counting with patient-reported appointments. Details informed by Radford *et al.* (2024).[11]

Table S4: Mean (sd) and mean (95% CI) difference in patient-reported community-based HCP appointments (NHS/PSS), by health-care professional type, over the 12-month treatment period (Participant reported, available case)

Resource item	Statistics	ESSVR + usual care (N=324)	Usual care (N=259)	Mean difference (95% CI)
Occupational therapist	Mean ± sd (n)	6.28 ± 7.76 (93)	4.75 ± 9.42 (63)	1.53 (-1.20 to 4.26)
General practitioner (GP)	Mean ± sd (n)	2.92 ± 3.01 (98)	2.35 ± 2.44 (62)	0.56 (-0.33 to 1.46)
Practice Nurse	Mean ± sd (n)	1.79 ± 2.95 (101)	1.02 ± 1.68 (64)	0.78 (-0.02 to 1.58)
District nurse	Mean ± sd (n)	0.39 ± 1.54 (101)	0.27± 1.03 (64)	0.12 (-0.31 to 0.55)
Counsellor	Mean ± sd (n)	0.48 ± 1.86 (101)	1.08 ± 2.56 (65)	-0.60 (-1.28 to 0.08)
SLT	Mean ± sd (n)	0.68 ± 2.44 (98)	1.23 ± 3.57 (65)	-0.55 (-1.48 to 0.38)
Physiotherapist	Mean ± sd (n)	4.98 ± 13.81 (97)	1.05 ± 2.37 (66)	3.93 (0.54 to 7.33)
Social worker	Mean ± sd (n)	0 ± 0 (101)	0.38 ± 2.26 (66)	-0.38 (-0.82 to -0.37)
Rehabilitation assistant	Mean ± sd (n)	1.31 ± 4.18 (98)	1.84 ± 10.28 (64)	-0.54 (-2.83 to 1.76)
Health care assistant	Mean ± sd (n)	1.45 ± 11.40 (99)	1.57 ± 11.79 (65)	-0.11 (-3.76 to 3.53)
Walk-in centre	Mean ± sd (n)	0.02 ± 0.20 (100)	0.06 ± 0.30 (66)	-0.04 (-0.12 to 0.04)
Other	Mean ± sd (n)	0.69 ± 2.08 (102)	0.71 ± 2.31 (65)	-0.02 (-0.70 to 0.66)

CI, confidence interval; sd, standard deviation; SLT, Speech and language therapist.
Available case data includes only those participants who completed the primary care section of the health resource use questionnaire at every follow-up period (0-3m, 3-6m and 6-12m).

Table S5: Mean (sd) and mean (95% CI) difference in patient-reported secondary care contacts, by visit type, over the 12-month treatment period (Participant reported, available case)

Resource item	Statistics	ESSVR + usual care (N=324)	Usual care (N=259)	Mean difference (95% CI)
Out-patient appointments				
Consultant	Mean ± sd (n)	1.80 ± 2.96 (94)	1.21 ± 1.45 (56)	0.58 (-0.25 to 1.42)
Occupational therapist	Mean ± sd (n)	0.92 ± 2.87 (101)	1.1 ± 3.58 (60)	-0.18 (-1.2 to 0.84)
Psychologist	Mean ± sd (n)	0.21 ± 0.89 (101)	0.57 ± 1.85 (61)	-0.37 (-0.79 to 0.06)
Physiotherapist	Mean ± sd (n)	1.97 ± 8.39 (101)	0.88 ± 3.19 (60)	1.09 (-1.14 to 3.32)
SLT	Mean ± sd (n)	0.19 ± 1.05 (101)	0.28 ± 1.10 (59)	-0.09 (-0.43 to 0.25)
Nurse	Mean ± sd (n)	0.54 ± 2.06 (101)	0.31 ± 0.98 (61)	0.23 (-0.32 to 0.79)
Stroke team	Mean ± sd (n)	1.41 ± 4.00 (101)	0.70 ± 1.17 (61)	0.70 (-0.33 to 1.73)
Radiology	Mean ± sd (n)	0.22 ± 0.53 (99)	0.15 ± 0.47 (62)	0.08 (-0.08 to 0.24)
Ophthalmology	Mean ± sd (n)	0.13 ± 0.50 (101)	0.13 ± 0.62 (60)	-0.00 (-0.18 to 0.17)
Cardiac & pulmonary tests	Mean ± sd (n)	0.25 ± 0.62 (101)	0.07 ± 0.31 (60)	0.18 (0.01 to 0.35)
Blood tests	Mean ± sd (n)	0.04 ± 0.24 (101)	0.03 ± 0.26 (60)	0.01 (-0.07 to 0.09)
Other	Mean ± sd (n)	0.34 ± 0.83 (101)	0.25 ± 1.22 (61)	-0.01 (-0.33 to 0.31)
In-patient stays				
Stroke ward	Mean ± sd (n)	0.63 ± 2.7 (104)	2.30 ± 9.76 (64)	-1.66 (-3.67 to 0.34)
Neuro/ stroke rehab unit	Mean ± sd (n)	0.41 ± 2.37 (104)	2.41 ± 14.9 (64)	-1.99 (-4.93 to 0.94)
ICU/HDU	Mean ± sd (n)	0 ± 0 (104)	0 ± 0 (62)	0 (-)
Other ward	Mean ± sd (n)	0.63 ± 3.21 (104)	1.55 ± 5.26 (64)	-0.92 (-2.21 to 0.37)

CI, confidence interval; HDU, high dependency unit; ICU, intensive care unit; sd, standard deviation; SLT, Speech and Language Therapist.

Available case data includes only those participants who completed the out-patient/in-patient section of the health resource use questionnaire at every follow-up period (0-3m, 3-6m and 6-12m).

Table S6: Logistic regression for missingness of costs and QALYs

	Odds ratio in logistic regression for missing data (95% CI)	
	**Missing data on costs**	**Missing data on QALYs**
Treatment allocation	1.80 (1.05 to 3.06)*	1.44 (0.95 to 2.17)
Age	0.99 (0.96 to 1.01)	0.99 (0.97 to 1.01)
Sex	1.16 (0.66 to 2.02)	0.84 (0.53 to 1.32)
Baseline utility score (EQ-5D)	0.77 (0.18 to 3.38)	0.74 (0.23 to 2.35)
EQ-5D mobility score at baseline	1.12 (0.80 to 1.56)	1.10 (0.85 to 1.42)
Stroke type: intracerebral haemorrhage	1.59 (0.14 to 17.62)	0.40 (0.04 to 4.06)
Stroke type: ischaemic	0.95 (0.09 to 9.51)	0.37 (0.04 to 3.60)
Living alone	0.99 (0.53 to 1.83)	1.19 (0.72 to 1.95)
Education level	1.26 (0.92 to 1.74)	1.37 (1.06 to 1.77)*
Comorbid cardiac complications	0.80 (0.40 to 1.57)	0.86 (0.50 to 1.46)
Comorbid mental health problems	1.05 (0.43 to 2.58)	0.70 (0.32 to 1.51)
Comorbid seizures	0.63 (0.07 to 5.66)	0.61 (0.11 to 3.29)
Comorbid musculoskeletal conditions	1.27 (0.65 to 2.46)	1.44 (0.83 to 2.52)
Comorbid diabetes	1.49 (0.78 to 2.87)	0.92 (0.52 to 1.62)
Site 10851	3.35 (0.94 to 11.94)	3.17 (1.16 to 8.67)*†

CI, confidence interval; QALYs, quality adjusted life-years
* Statistical significance at 0.05
† No significant association with missingness was found for other sites

Figure S1: Formulae for estimating medication costs

BL_med_tot = BL_med x 1.5

3M_med_tot = (BL_med_tot x 2) - (3M_med_stop x 3) + (3M_med_start x 3)

6M_med_tot = 3M_med_tot - (6M_med_stop x 3) + (6M_med_start x 3)

12M_med_tot = (6M_med_tot x 2) - (12M_med_stop x 6) + (12M_med_start x 6)

BL_med: baseline medications with PCA 'cost per item' applied.
3M_med_stop/3M_med_start: 3 month stop/start medications with PCA 'cost per item' applied.
6M_med_stop/6M_med_start: 6 month stop/start medications with PCA 'cost per item' applied.
12M_med_stop/12M_med_start: 12 month stop/start medications with PCA 'cost per item' applied.
BL_med_tot: Total per-participant baseline medication cost
3M_med_tot: Total per-participant 3-month medication cost
6M_med_tot: Total per-participant 6-month medication cost
12M_med_tot: Total per-participant 12-month medication cost

References

1. Jones K, Weatherly H, Castelli A, et al. Unit Costs of Health and Social Care 2022 Manual, https://www.pssru.ac.uk/unitcostsreport/ (2022, accessed 22 December 2023).
2. NHS England. National Cost Collection for the NHS, https://www.england.nhs.uk/costing-in-the-nhs/national-cost-collection/ (2022, accessed 22 December 2023).
3. Curtis L, Burns A. Unit costs of health and social care 2015. Personal Social Services Research Unit, University of Kent, Canterbury, 2015.
4. Curtis L. Unit costs of health and social care 2010. Personal Social Services Research Unit, University of Kent, Canterbury, 2010.
5. Jones K, Burns A. Unit Costs of Health and Social Care 2021. PSSRU, https://www.pssru.ac.uk/project-pages/unit-costs/unit-costs-of-health-and-social-care-2021/ (2021, accessed 5 December 2022).
6. UKHCA. UKHCA Commisioning Survey 2012: Care is not a Commodity, file://ueahome/eresfmh4/jry14qdu/data/Downloads/UKHCACommissioningSurvey2012.pdf (2012, accessed 7 August 2023).
7. NHS Business Services Authority. Prescription Cost Analysis - England - 2021/22, https://www.nhsbsa.nhs.uk/statistical-collections/prescription-cost-analysis-england/prescription-cost-analysis-england-202122 (2022, accessed 17 May 2024).
8. Radford K, Sutton C, Sach T, et al. Early, specialist vocational rehabilitation to facilitate return to work after traumatic brain injury: The FRESH feasibility RCT. Health Technol Assess (Rockv) 2018; 22: 1–123.
9. Office for National Statistics. Annual Survey of Hours and Earnings time series of selected estimates, https://www.ons.gov.uk/employmentandlabourmarket/peopleinwork/earningsandworkinghours/datasets/ashe1997to2015selectedestimates (2022, accessed 6 March 2023).
10. Gov.uk. Benefits and financial support if you’re disabled or have a health condition, https://www.gov.uk/browse/benefits/disability (2024, accessed 3 July 2024).
11. Radford K, Grant M, Holmes J, et al. Development and description of the Early Stroke Specialist Vocational Rehabilitation (ESSVR) intervention delivered in the Return to work after stroke (RETAKE)Trial. Submitted to Health Technology Assessment.
