# Supplementary material for: Cost consequences analysis of early vocational rehabilitation compared with usual care for stroke survivors
Source: Clin Rehabil. 2024 Dec 5;39(2):161–73. doi: 10.1177/02692155241299372 (PMC11846267; doi:10.1177/02692155241299372)
Supplement: sj-pdf-2-cre-10.1177_02692155241299372 - Supplemental material for Cost consequences analysis of early vocational rehabilitation compared with usual care for stroke survivors [file sj-pdf-2-cre-10.1177_02692155241299372.pdf]

Supplementary material 2: Carer resource use, costs and QALYs

Methods

Participants could choose to nominate a carer (someone who provides support at least once per week) or not to participate in the trial. Participant-nominated, consented carers were asked to complete a questionnaire booklet, with resource use questions relating to their own use of services and EQ-5D-5L questionnaire to be completed about their own health-related quality-of-life, at baseline, three, six and 12 months. The aim was to explore whether receiving Early Stroke Specialist Vocational Rehabilitation (ESSVR) or not had a knock-on impact on the level of resources used and health-related quality of life of the nominated carer. We also asked participants to report if their family or friends incurred any out-of-pocket costs or time off work and these are reported within the main paper. Only carer-reported data is included in this section of the supplementary material.

Estimating health-related quality-of-life and quality-Adjusted Life years (QALYs) for the participant-nominated carer over the 12-month study period was undertaken using the same approach as that outlined in the main paper for participants.

Results

Just under a third of participants identified a carer and carers were recruited for 137 (23.5%) participants (n=70 for ESSVR and n=65 for usual care).

As with participants, data completion declined over the course of the study. In terms of complete EQ-5D-5L data at all four timepoints (such that a 12-month QALY score could be

estimated) it was only available for 14 (20%) of ESSVR and 5 (8%) of usual care participant-nominated carers.

Table S7 presents the unadjusted mean (sd) outcomes and mean (95% CI) difference in outcomes over 12 months, using available case data, for nominated carers. Utility scores are similar between groups at all timepoints, but carer numbers are low with only 135/583 participants nominating carers, and complete case data available for only 85/135 (14.6% of 583 participants).

Conclusion

Given the small proportion of participant-nominated carers and the levels of missing data it is hard to draw any conclusion about whether there was any knock-on impact for carers of ESSVR.

Table S7: Carer mean (sd) and mean (95% CI) difference in outcomes over 12 months (available case analysis)

Carers	ESSVR + UC (N=70)	UC (N=65)	
Utility score (EQ-5D-5L) at baseline	0.828 ± 0.178 (65)	0.863 ± 0.128 (63)	-0.035 (-0.090 to 0.019)
Utility score (EQ-5D-5L) at 3 months	0.841 ± 0.124 (28)	0.885 ± 0.129 (18)	-0.043 (-0.120 to 0.033)
Utility score (EQ-5D-5L) at 6 months	0.829 ± 0.169 (27)	0.882 ± 0.126 (10)	-0.052 (-0.171 to 0.067)
Utility score (EQ-5D-5L) at 12 months	0.841 ± 0.118 (18)	0.876 ± 0.125 (5)	-0.035 (-0.161 to 0.091)
12-month QALY score	0.848 ± 0.089 (14)	0.865 ± 0.128 (5)	-0.018 (-0.127 to 0.092)

CI, confidence interval; UC, usual care
