# Supplementary material for: Cost consequences analysis of early vocational rehabilitation compared with usual care for stroke survivors
Source: Clin Rehabil. 2024 Dec 5;39(2):161–73. doi: 10.1177/02692155241299372 (PMC11846267; doi:10.1177/02692155241299372)
Supplement: sj-pdf-3-cre-10.1177_02692155241299372 - Supplemental material for Cost consequences analysis of early vocational rehabilitation compared with usual care for stroke survivors [file sj-pdf-3-cre-10.1177_02692155241299372.pdf]

ISRCTN12464275

RETAKE

Participant Questionnaire Pack • 3 Months

To be completed by the researcher:

Participant Initials		Date of Birth	Day / Month / Year	Participant ID	Site Code / Trial No
Date sent	Day / Month / Year	OR	Researcher/Therapist name *(If completed by phone or face-to-face)*		

RETAKE – RETurn to work After stroKE

We would be grateful if you could complete the following questionnaires to help us find out more about you and your experiences and abilities after your stroke.

Even if you have not returned to work or are not receiving support from services after discharge from hospital, you are still a very important part of this study. We are interested in every stroke survivor taking part in the study.

We will use this information for research purposes only and there will be no change to your clinical care. If you have any concerns about your care you should contact your GP or a member of your healthcare team.

All your answers will remain **completely confidential**.

Step 1. The questionnaire is divided into 6 sections. Please read the instructions at the beginning of each section. If you need to complete the booklet over more than one day, please do so.

If you have any difficulty completing the questionnaire, someone can help you by reading the questions and/or completing the answers. If you are able, you must think of your own responses.

There are no right or wrong answers to any of the questions; your answers should just reflect how you feel and your own experience.

Step 2. If you are unable or do not wish to complete parts of this questionnaire you do not have to. Please complete as much as you can.

Step 3. Please return the completed pack to us in the pre-paid envelope provided.

If you have any questions or would like help to complete this questionnaire, please contact the RETAKE team at: RETAKE@leeds.ac.uk or contact us on: 0115 823 0315.

Thank you for your time and valuable contribution to this study.

Dr. Kathryn Radford, Lead Researcher

University of Nottingham

The University of Nottingham

ctru University of Leeds

UNIVERSITY OF LEEDS

Contents

Please use the checklist below to keep track of where you are up to in this questionnaire booklet – Remember you can take a break at any time and come back another time.

Section	Page	Section complete?
1: Work Status	1 – 4	☐
2: Work Ability	5	☐
3: Health-related Quality of Life	6	☐
4: Hospital Anxiety and Depression Scale	7	☐
5: Health Services Received	8 – 19	☐
6: Help Questions	20	☐

Section 1: Work Status

In this section we are trying to find out about your current work or educational status and the impact of your stroke on what you are doing now. Please tick the answers that best describe your situation.

Please write today's date: …………/…………../………….. (day / month / year)

Question 1:

Are you currently in work (paid or unpaid) for at least 2 hours per week?

☐ Yes

☐ No

Please go to question 2

Question 2:

What is your current main employment status (where employment includes unpaid and voluntary roles)?

Please tick one:

☐ Paid employment (including sick leave)

☐ Self-employment

☐ Voluntary work

☐ Education/studying

☐ Unemployed

☐ Retired

☐ Housework

☐ Other: Please specify ________________________________

Please go to question 3

Question 3:

In the last 3 months, has your stroke had an impact on your employment status reported in question 2?

☐ No, please go to question 4.

☐ Yes

If yes, please complete the table below:

Remember we are only interested in changes that have occurred as a result of your stroke.

I have had to take time off (e.g. sick leave, paid/unpaid leave)<br>☐ No<br>☐ Yes	If yes, how much time have you taken off in the last 3 months?<br>____________weeks<br>If you are in paid employment or self-employment was this leave paid?<br>☐ Yes ☐ No ☐ Mixture of paid and unpaid leave<br>If a mixture of paid and unpaid leave, how much of the leave was paid leave?<br>____________weeks
I have changed the hours I work each week<br>☐ No<br>☐ Yes	If yes, how have your work hours changed?<br>☐ I have decreased the hours I work each week<br>☐ I have increased the hours I work each week<br>☐ I have completely stopped work and will not be going back to it<br>How many hours per week do you work now?<br>____________hours ☐ N/A<br>How long ago did this change?<br>____________weeks

I have changed my role at work<br>☐ No<br>☐ Yes	If yes, what was your old job title:<br>________________________________<br>What is your new job title:<br>________________________________<br>How long ago did this change?<br>____________weeks
If you are still in work have you had to take more breaks than usual?<br>☐ No<br>☐ Yes<br>☐ N/A	If yes, how long in total have your additional breaks been in a typical week?<br>____________hours ____________Minutes

Question 4:

In the last 3 months, have you applied and/or received any state benefits?

(E.g. incapacity benefit/employment and support allowance (ESA), disability living allowance/personal independence payment (PIP), income support etc.)

☐ No, please go to question 5.

☐ Yes

If yes, please list any benefits you have applied for and/or received:

____

____

____

____

____

Do you rely on these benefits as a sole source of income?

☐ No

☐ Yes

Question 5:

If you are back at work, have you had a work accident in the last 3 months?

We are asking this question to find out about the safety of returning to work after stroke.

- ☐ No, please go to question 6.
- ☐ Yes
- ☐ Not applicable

If yes, please describe the accident(s):

____

____

____

____

____

Question 6:

If appropriate, in the last 3 months has your employer incurred any costs (e.g. for specialist equipment or adaptation at work) to support you in work?

- ☐ No
- ☐ Yes
- ☐ Not known
- ☐ Not applicable

If yes, please provide a description:

____

____

____

____

____

You have now completed section 1 of 6.

NEED A REST? It’s OK to have a break and come back another time.

Section 2: Work Ability

Assume that your work ability at its best has a value of 10 points. How many points would you give your current work ability?

(0 means that you currently cannot work at all)

0 1 2 3 4 5 6 7 8 9 10

Completely unable to work — Work ability at its best

You have now completed section 2 of 6.
NEED A REST? It's OK to have a break and come back another time.

Section 3: Health-related Quality of Life

The questions below help us find out about your health and well-being since your stroke.

Under each heading, please tick the ONE box that best describes your health TODAY.

MOBILITY

I have no problems in walking about ❑

I have slight problems in walking about ❑

I have moderate problems in walking about ❑

I have severe problems in walking about ❑

I am unable to walk about ❑

SELF-CARE

I have no problems washing or dressing myself ❑

I have slight problems washing or dressing myself ❑

I have moderate problems washing or dressing myself ❑

I have severe problems washing or dressing myself ❑

I am unable to wash or dress myself ❑

USUAL ACTIVITIES *(e.g. work, study, housework, family or leisure activities)*

I have no problems doing my usual activities ❑

I have slight problems doing my usual activities ❑

I have moderate problems doing my usual activities ❑

I have severe problems doing my usual activities ❑

I am unable to do my usual activities ❑

PAIN / DISCOMFORT

I have no pain or discomfort ❑

I have slight pain or discomfort ❑

I have moderate pain or discomfort ❑

I have severe pain or discomfort ❑

I have extreme pain or discomfort ❑

ANXIETY / DEPRESSION

I am not anxious or depressed ❑

I am slightly anxious or depressed ❑

I am moderately anxious or depressed ❑

I am severely anxious or depressed ❑

I am extremely anxious or depressed ❑

You have now completed section 3 of 6.

NEED A REST? It's OK to have a break and come back another time.

UK (English) © 2009 EuroQol Group EQ-5D™ is a trade mark of the EuroQol Group

Section 4: Hospital Anxiety and Depression Scale

Clinicians are aware that emotions play an important part in most illnesses. If your clinician knows about these feelings he or she will be able to help you more.

This questionnaire is designed to help your clinician to know how you feel. Read each item below and **tick the box** which comes closest to how you have been feeling in the past week.

Don't take too long over your replies, your immediate reaction to each item will probably be more accurate than a long, thought-out response.

I feel tense or 'wound up'
- ☐ Most of the time
- ☐ A lot of the time
- ☐ From time to time, occasionally
- ☐ Not at all

I still enjoy the things I used to enjoy
- ☐ Definitely as much
- ☐ Not quite so much
- ☐ Only a little
- ☐ Hardly at all

I get a sort of frightened feeling as if something awful is about to happen
- ☐ Very definitely and quite badly
- ☐ Yes, but not too badly
- ☐ A little, but it doesn't worry me
- ☐ Not at all

I can laugh and see the funny side of things
- ☐ As much as I always could
- ☐ Not quite so much now
- ☐ Definitely not so much now
- ☐ Not at all

Worrying thoughts go through my mind
- ☐ A great deal of the time
- ☐ A lot of the time
- ☐ Not too often
- ☐ Very little

I feel cheerful
- ☐ Never
- ☐ Not often
- ☐ Sometimes
- ☐ Most of the time

I can sit at ease and feel relaxed
- ☐ Definitely
- ☐ Usually
- ☐ Not often
- ☐ Not at all

I feel as if I am slowed down
- ☐ Nearly all the time
- ☐ Very often
- ☐ Sometimes
- ☐ Not at all

I get a sort of frightened feeling like 'butterflies' in the stomach
- ☐ Not at all
- ☐ Occasionally
- ☐ Quite often
- ☐ Very often

I have lost interest in my appearance
- ☐ Definitely
- ☐ I don't take as much care as I should
- ☐ I may not take quite as much care
- ☐ I take just as much care as ever

I feel restless as if I have to be on the move
- ☐ Very much indeed
- ☐ Quite a lot
- ☐ Not very much
- ☐ Not at all

I look forward with enjoyment to things
- ☐ As much as I ever did
- ☐ Rather less than I used to
- ☐ Definitely less than I used to
- ☐ Hardly at all

I get sudden feelings of panic
- ☐ Very often indeed
- ☐ Quite often
- ☐ Not very often
- ☐ Not at all

I can enjoy a good book or radio or television programme
- ☐ Often
- ☐ Sometimes
- ☐ Not often
- ☐ Very seldom

You have now completed section 4 of 6.
NEED A REST? It's OK to have a break and come back another time.

HADS copyright © R.P. Snaith and A.S. Zigmond, 1983, 1992, 1994. Record form items originally published in Acta Psychiatrica Scandinavica, 67, 361–70, copyright © Munksgaard International Publishers Ltd, Copenhagen, 1983. This edition first published in 1994 by nferNelson Publishing Company Ltd, now GL Assessment Limited, 1st Floor Vantage London, Great West Road, Brentford TW8 9AG United Kingdom. GL Assessment is part of GL Education www.gl-assessment.co.uk. This form may not be reproduced by any means without first obtaining permission from the publisher. Email: permissions@gl-assessment.co.uk. All rights reserved including translations

Section 5: Health Services Received

Question 1: Health problems

Have you been diagnosed with any new health problems in the last 3 months?

☐ No, please go to question 2a

☐ Yes

If yes, please specify the health problem below:

☐ Cardiac complications

☐ Mental health problems

☐ Seizures

☐ Musculoskeletal conditions

☐ Diabetes

☐ Other, please specify

____

With these next few questions, we are trying to find out more about the services you have received since your stroke and as a result of your stroke.

Please include anything which you consider to be related to having had a stroke. For instance, you may feel another condition such as a musculoskeletal or a mental health condition has arisen directly as a result of experiencing a stroke – this should be included. Please also include any care received as a result of adverse reactions to medications you may be taking as a result of having had a stroke.

Question 2: Hospital-based services

If you are unsure, please put in your best estimate.

2a. **In the last 3 months**, have you been admitted to hospital as an inpatient as a result of your stroke?

☐ No, please go to question 2b

☐ Yes, please complete the table below:

If yes, for **each inpatient** visit you have had, please record the type of ward you were admitted to and the duration of your stay.

Please include any day case procedures.

Visit number	The type of department or ward or reason for admission	Duration of each stay (in days)	
Example	*Neurology*	*2*	days
1			days
2			days
3			days
4			days

2b. **In the last 3 months** have you visited a hospital as an outpatient as a result of your stroke?

☐ No, please go to question 2c

☐ Yes, please complete the table below:

If yes, for **each outpatient** visit you had at the hospital as a result of your stroke, please record the type of professional you saw and the number of visits.

If you are unsure, please put in your best estimate.

Please do not include visits with any of these professionals that took place outside of the hospital. These should be included in question 3 below.

**Professional person you saw** *(If unknown, please write the department in which you saw them)*	**Number of outpatient visits**
Example: Consultant neurologist	*2* visits
Consultant, please state hospital department seen in: ____	visits
Psychologist	visits
Physiotherapist	visits
Speech and language therapist (SLT)	visits
Nurse	visits
Stroke team	visits
Occupational therapist (OT)	visits
Other, please specify: ____________________________________	visits
Other, please specify: ____________________________________	visits
Other, please specify: ____________________________________	visits

2c. Did you attend Accident and Emergency Services **for any reason** in the last 3 months?

☐ No, please go to question 3

☐ Yes

If yes, please state how many visits in the last 3 months: ______________________

Question 3: Community-based NHS services or social services

3. In the last 3 months have you visited, or been visited, by any health or social care professional in a community setting (e.g. at a GP practice or home) as a result of your stroke?

☐ No, please go to question 4

☐ Yes, please complete the table below:

If you are receiving the trial vocational rehabilitation intervention, please include the visits received as part of this in your answers below.

For each professional listed, please mark 'No' if you did not see them at all or 'Yes' if you saw them and enter the number of visits.

GP	☐ No	☐ Yes	If yes, how many times?	________
Practice nurse (typically seen at GP surgery)	☐ No	☐ Yes	If yes, how many times?	________
District nurse (typically seen in your own home)	☐ No	☐ Yes	If yes, how many times?	________
Counsellor	☐ No	☐ Yes	If yes, how many times?	________
Occupational therapist	☐ No	☐ Yes	If yes, how many times?	________
Speech and language therapist	☐ No	☐ Yes	If yes, how many times?	________
Physiotherapist	☐ No	☐ Yes	If yes, how many times?	________
Social worker	☐ No	☐ Yes	If yes, how many times?	________
Rehabilitation Assistant (e.g. someone who supports your physical recovery)	☐ No	☐ Yes	If yes, how many times?	________
Health care assistant (e.g. someone who helps with personal care)	☐ No	☐ Yes	If yes, how many times?	________
NHS walk in centre	☐ No	☐ Yes	If yes, how many times?	________
Other, please specify who: ______________________________	☐ No	☐ Yes	If yes, how many times?	________
Other, please specify who: ______________________________	☐ No	☐ Yes	If yes, how many times?	________

Question 4: Medication

4a. In the last 3 months have you been **prescribed** any **<u>new</u>** medications?

☐ No, please go to question 4b

☐ Yes

If yes, please specify the name of the medication and how many prescriptions of this you have had.

**Name of prescribed medication**	**How many prescriptions of this have you had?**
Example: Panadol Advance Tablet 500 mg	*2*

4b. In the last 3 months have you **stopped** taking any **prescribed** medications?

☐ No, please go to question 5

☐ Yes

If yes, please specify the name of the medication and how many prescriptions you had before stopping.

**Name of prescribed medication**	**How many prescriptions of this have you had?**
Example: Panadol Advance Tablet 500 mg	*2*

Question 5: Equipment

5. In the last 3 months have you been given any equipment to help you cope with your stroke? *(Please do not list any you have purchased yourself here).*

☐ No, please go to question 6

☐ Yes

If yes, please list the items purchased for you by the NHS or social services *(for example; walking aids e.g. stick, frame, wheelchair; hoists; bath aids; kitchen aids e.g. plates, cutlery, jar opener).*

____

____

____

Question 6: Employment services

In the last 3 months please tell us if you have had contact with any of the people listed below:

Benefits advisor	☐ No	☐ Yes	If yes, how many times? ________
Mandatory visits to the job centre	☐ No	☐ Yes	If yes, how many times? ________
Other services aimed at helping you get or stay in work<br>If yes, please give details of the service(s):<br>______________________<br>______________________	☐ No	☐ Yes	If yes, how many times? ________
Have you seen anyone else who has helped you?<br>If yes, please give details:<br>______________________<br>______________________	☐ No	☐ Yes	If yes, how many times? ________

Question 7: Other services

In the last 3 months have you received any other services due to your stroke?

For example, early supported discharge service.

☐ No, please go to question 8

☐ Yes

If yes, please give details including type and how many times received:

Other service received / used (due to your stroke)	Number of times received / used

Costs incurred by yourself or family

With these next few questions, we are trying to find out more about the costs incurred by you and your family/friends in addition to the care your family and friends have provided you over the last 3 months. This information will remain confidential.

Question 8: Personal Costs

8a. In the **last 3 months** have you or your family/friends incurred any other costs as a result of your stroke (e.g. complementary therapists, equipment, non-prescribed medication, travel or parking for attending health care appointments, income protection insurance etc.)?

☐ No, please go to question 8b

☐ Yes

If yes, please state the details and the approximate cost.

For example, 1 visit to a private physiotherapist cost £50 (1 × £50); 2 visits to an acupuncturist, total cost £80 (2 × £40).

**Item**	**Number of items or visits**	**Overall cost (£)**
Example: Acupuncturist	*2*	*£80*
Example: Private physiotherapist	*1*	*£50*
Complementary therapists (e.g. aromatherapist, acupuncturist, hydrotherapy, homeopathy etc.)		£
Non-prescribed medication		£
Travel costs to health care appointments		£
Parking costs at health care appointments		£
Income protection insurance		£
Equipment		£
Other, please specify ______________________		£
Other, please specify ______________________		£
Other, please specify ______________________		£

8b. In the **last 3 months** have you had to pay someone, who is **not** a friend or relative, to do shopping, cleaning or personal care tasks for you?

☐ No, please go to question 9

☐ Yes

If yes, how much did you spend in a typical week on such help?

£___________ per week

Question 9: Support

9a. In the **last 3 months** has a friend or relative changed their paid working hours as a result of your stroke (e.g. to help look after you or support you financially)?

☐ No, please go to 9c

☐ Yes

If yes, please complete the table below:

They have had to give up paid work completely<br>☐ No<br>☐ Yes	If yes, how many hours did they typically work per week before giving up paid work?<br>_________hours<br>And how many weeks has it been since they gave up paid work?<br>_________weeks
They have taken time off<br>☐ No<br>☐ Yes	If yes, how much time have they taken off in the last 3 months? (complete as appropriate)<br>____________months ____________weeks<br>____________days ____________hours
They have changed their paid work hours<br>☐ No<br>☐ Yes	If yes, how have their paid work hours changed in the last 3 months?<br>☐ They have decreased their hours<br>How many hours of paid work have they given up in a typical week to care for you?<br>____________hours<br>Or<br>☐ They have increased their hours<br>How much more paid work have they undertaken in a typical week?<br>____________hours

9b. Is this friend or relative the same one you have nominated to complete a separate carer questionnaire?

☐ No, please go to question 9c

☐ Yes

If yes, do you have any other friends or relatives who have changed their paid work hours as a results of your stroke (e.g. to help look after you or support you financially)?

☐ No, please go to question 9c

☐ Yes

If yes, please complete the table below:

How many relatives / friends have changed their paid working hours as a result of your stroke?<br>_________________number of relatives / friends	
How has your relative/friend's paid work changed?	
They have had to give up work completely<br>☐ No<br>☐ Yes	If yes, how many hours did they typically work per week before giving up paid work (collectively)?<br>_________hours<br>And how many weeks has it been since they gave up paid work (collectively)?<br>_________weeks
They have taken time off<br>☐ No<br>☐ Yes	If yes, how much time have they taken off in the last 3 months (collectively)? (complete as appropriate)<br>____________months ____________weeks<br>____________days ____________hours
They have changed their paid work hours<br>☐ No<br>☐ Yes	If yes, how have their paid work hours changed in the last 3 months?<br>☐ They have decreased their hours<br>How many hours of paid work have they given up in a typical week to care for you (collectively)?<br>____________hours<br>Or<br>☐ They have increased their hours<br>How much more paid work have they undertaken in a typical week (collectively)?<br>____________hours

9c. **Support outside of official services** (For example, charity support groups such as stroke association support groups, helplines etc.)

In the **last 3 months**, have you received support or attended support groups as a result of your stroke?

☐ No, please go to Section 6.

☐ Yes

If yes, please list what support you have accessed and state whether you incurred any costs as a result (e.g. membership fee, participation fee, telephone cost etc.)

**Type of Support**	**Cost Incurred (£)**
	£
	£
	£
	£
	£
	£
	£

You have now completed section 5 of 6.

NEED A REST? It's OK to have a break and come back another time.

Section 6: Help Questions

1) **Did anyone help you to complete this questionnaire?**

No ☐ **If no**, please go to the next page

Yes ☐ **If yes**, please answer the questions below

If someone helped you to complete the questionnaire:

1a) How were you helped? ***(Please tick as many as appropriate)***

- Someone read out the questions ☐
- Someone translated the questions (if English is not your first language) ☐
- Someone discussed the questions with you ☐
- Someone ticked the boxes ☐
- All questionnaires were completed on the person's behalf without consulting them ☐
- Other type of help. Please specify below: ☐

………………………………………………………………………………….

………………………………………………………………………………….

1b) **Please place a tick in the box which best describes the relationship between you and the person who helped you.**

The person who helped you is your…

- Partner (married / never married / divorced / separated) ☐
- Daughter / Son (including in-law, step-child) ☐
- Grandchild ☐
- Other relative ☐
- Friend / neighbour ☐
- Paid carer ☐
- Nurse ☐
- Health visitor ☐
- Rehabilitation assistant ☐
- Other non-relative ☐
- RETAKE researcher ☐

You have now completed the questionnaire. Thank you.

Please take a moment to look through the questionnaire booklet and check that you have answered all questions. Then return the completed booklet, in the envelope provided, to us at the following address:

RETAKE Study Team
Clinical Trials Research Unit (CTRU)
University of Leeds
Leeds
LS2 9JT

This study is funded by the National Institute for Health Research (NIHR) HTA Programme (Project number: 15/130/11). The views expressed are those of the author(s) and not necessarily those of the NIHR or the Department of Health and Social Care.

FUNDED BY
NIHR | National Institute for Health Research

For office use only			
Computerised		Verified/Checked	
Date	Initials	Date	Initials
