# Supplementary material for: Cost consequences analysis of early vocational rehabilitation compared with usual care for stroke survivors
Source: Clin Rehabil. 2024 Dec 5;39(2):161–73. doi: 10.1177/02692155241299372 (PMC11846267; doi:10.1177/02692155241299372)
Supplement: sj-pdf-4-cre-10.1177_02692155241299372 - Supplemental material for Cost consequences analysis of early vocational rehabilitation compared with usual care for stroke survivors [file sj-pdf-4-cre-10.1177_02692155241299372.pdf]

<u>RETAKE Research Group</u>

Members of the RETAKE Research Group (bold included as authors)

Prof Kate Radford
Prof Amanda Farrin
Prof Dame Caroline Watkins
Prof Rory J O'Connor
Prof Tracey Sach
Sarah Pyne
Helen Risebro
Dr Rory Cameron
Prof Audrey Bowen
Dr David J Clarke
Dr Katie Powers
Alexandra Wright-Hughes
Ellen Thompson
Florence Day
Dr Diane Trusson
Kristelle Craven

Former members:

Vicki Mclellan
Suzanne Hartley
Ivana Holloway
Bonnie Cundill
Sara Clarke
Prof Marion Walker

Patient and Public Involvement Partners (PPI)

Prof Christopher McKevitt
Dr Judith Stevens
John Murray
Margaret Cheng
Tony Boyce
Isabella Iyama
Martin Coult

OT Mentors

Dr Julie Phillips
Dr Jain Holmes
Ruth Tyerman
Yash Bedekar
Jo Hurford
