# Supplementary material for: Cost consequences analysis of early vocational rehabilitation compared with usual care for stroke survivors
Source: Clin Rehabil. 2024 Dec 5;39(2):161–73. doi: 10.1177/02692155241299372 (PMC11846267; doi:10.1177/02692155241299372)
Supplement: sj-docx-6-cre-10.1177_02692155241299372 - Supplemental material for Cost consequences analysis of early vocational rehabilitation compared with usual care for stroke survivors [file sj-docx-6-cre-10.1177_02692155241299372.docx]

# Supplementary material: Contents

Supplementary material 1
**Title**: Additional Tables and Figures
**Description**: Contains additional tables and figures, referred to in the main manuscript and giving further detailed analysis.
**File**: Supp1_Additional_Tables_&_Figures.pdf

Supplementary material 2
**Title**: Carer resource use, costs and QALYs
**Description**: Describes the methods, results and conclusions for an additional analysis of resource use, costs and quality-adjusted life-years (QALYs) of carers nominated by participant stroke-survivors.
**File**: Supp2_Carer_Costs_&_QALYs.pdf

Supplementary material 3
**Title**: Example resource-use questionnaire (3 months)
**Description**: An example of the patient-reported resource use questionnaire used at the 3-month timepoint. Very similar questionnaires were also used at baseline, 6 and 12 months.
**File**: Supp3_Resource-use_Questionnaire.pdf

Supplementary material 4
**Title**: RETAKE Research Group

**Description**: Complete list of all members of the RETAKE Research Group.
**File**: Supp4_RETAKE_research_grp.pdf
